# Thyroid Eye Disease in the Teprotumumab Era: A Narrative Review of Expanding the Therapeutic Window Beyond Disease Activity

**DOI:** 10.7759/cureus.116558

**Published:** 2026-09-19

**Authors:** Zubaida Sirang, Raisa Khan, Wardah Moazzum, Irfan Jeeva

**Affiliations:** 1 Department of Ophthalmology, Oculofacial Plastic and Orbital Surgery, University of Calgary, Calgary, CAN; 2 Department of Ophthalmology and Visual Sciences, Aga Khan University Hospital, Karachi, PAK; 3 Department of Pharmacy, Aga Khan Health Service, Pakistan, Karachi, PAK; 4 Department of Ophthalmology, Mid Yorkshire Teaching NHS Trust, Wakefield, GBR

**Keywords:** clinical activity score, graves’ orbitopathy, immunosuppressive therapy, mycophenolate, orbital decompression, selenium, ted, teprotumumab, thyroid eye disease

## Abstract

Thyroid eye disease (TED) is an autoimmune inflammatory disorder most commonly associated with Graves' disease and encompasses a broad clinical spectrum, ranging from mild eyelid and ocular surface changes to sight-threatening complications such as dysthyroid optic neuropathy (DON). This narrative review provides an updated, clinically oriented synthesis of TED, emphasizing the distinction between disease activity and severity, contemporary clinical assessment, established treatment strategies, and the evolving therapeutic window of targeted therapy.

PubMed/Medical Literature Analysis and Retrieval System Online (MEDLINE) was searched, with the literature supplemented by targeted review of contemporary guidelines, consensus statements, pivotal clinical trials, prospective studies, systematic reviews, and reference lists of key publications. Particular emphasis was placed on disease activity and severity, conventional immunomodulatory treatment, teprotumumab, emerging insulin-like growth factor-1 receptor (IGF-1R)-directed therapies, and evidence addressing longstanding or less-active disease, delayed response, retreatment, durability, and reactivation.

The Clinical Activity Score (CAS) and Vision, Inflammation, Strabismus, and Appearance (VISA) framework provide structured approaches to assessing disease activity and clinical manifestations. Management is guided by disease activity, severity, disease duration, phenotype, and treatment goals, ranging from supportive measures, risk-factor modification, and selenium supplementation in appropriate patients with mild disease to intravenous glucocorticoids, mycophenolate, targeted therapy, and selected orbital radiotherapy in active moderate-to-severe disease. Teprotumumab has broadened therapeutic options beyond the traditional paradigm of treating predominantly early, highly active TED, with emerging evidence supporting clinically meaningful responses in longstanding and less-active disease, including delayed responses and retreatment. Long-term studies also highlight variability in durability and the possibility of disease reactivation. Veligrotug and other targeted approaches are further expanding the therapeutic landscape. Sight-threatening disease requires urgent treatment, whereas rehabilitative orbital, strabismus, and eyelid surgery remain central for persistent structural sequelae. Overall, contemporary TED management is shifting from a predominantly phase-based model toward individualized treatment based on activity, severity, disease duration, phenotype, and the patient's principal therapeutic goal. Important questions regarding durability, retreatment, long-term safety, predictors of response, cost, and access remain unresolved.

## Introduction and background

Thyroid eye disease (TED), also known as thyroid-associated orbitopathy (TAO), can significantly impact appearance and vision. This autoimmune disorder is the most frequent extrathyroidal manifestation of Graves’ disease and is the most common orbital disease affecting adults in many developed countries [1,2]. Epidemiological estimates vary across populations and study designs, and clinically apparent TED occurs in a substantial proportion of patients with Graves’ disease [1,2]. The disorder is more commonly seen in women, whereas men often experience more severe disease [1]. TED usually arises with hyperthyroidism, yet it can also develop in euthyroid and hypothyroid states, including Hashimoto thyroiditis [3].

The chance and impact of disease tend to increase with age, especially among men and smokers. Smoking is the most consistently reported modifiable risk factor and is associated with a longer and more severe disease course [1,4]. TED may occur before, with, or after the onset of thyroid dysfunction, and orbital manifestations may initially be subtle [1,2].

TED has a broad clinical spectrum, ranging from ocular-surface symptoms and eyelid changes to diplopia, marked proptosis, exposure keratopathy, and dysthyroid optic neuropathy (DON), which can cause permanent visual impairment or vision loss [1,5]. In addition to physical effects, TED can substantially affect psychosocial well-being and quality of life [6,7].

Disease activity and disease severity are related but distinct concepts. Activity reflects ongoing inflammatory activity and the likelihood that a patient may respond to immunosuppressive or targeted anti-inflammatory treatment. Severity reflects the functional and anatomical burden of disease, including proptosis, diplopia, corneal exposure, optic nerve dysfunction, and impact on daily life. TED commonly evolves from an active inflammatory phase into an inactive residual phase, although the duration and trajectory vary among patients [1,2]. This distinction is central to treatment selection because active disease may be amenable to medical therapy, whereas persistent structural sequelae in inactive disease often require rehabilitative surgery.

The management of TED has historically focused on corticosteroid therapy, other immunomodulatory approaches, and surgery, with disease activity serving as an important determinant of candidacy for medical treatment. Advances in understanding thyroid-stimulating hormone receptor (TSHR) and insulin-like growth factor-1 receptor (IGF-1R) signaling, orbital fibroblast biology, and immune-cell interactions have expanded therapeutic strategies [8-10]. In particular, the introduction of teprotumumab, an IGF-1R-directed monoclonal antibody, has represented a major shift toward mechanism-based treatment. Its clinical development has also challenged the traditional assumption that meaningful therapeutic benefit is restricted to highly active or early TED, as subsequent evidence has demonstrated benefit in patients with longer-duration and relatively low-activity disease [11-13].

This review was undertaken to provide an updated, clinically oriented synthesis of TED assessment and treatment, with particular emphasis on the distinction between activity and severity and the implications of the expanding therapeutic window. The emergence of teprotumumab has made this distinction increasingly important: activity remains central to assessing inflammatory burden and treatment urgency, but it may no longer fully define whether clinically meaningful disease features are potentially treatable. Rather than providing equal-weight summaries of every available immunomodulator, this review examines how evidence for teprotumumab across active, longstanding, and retreatment settings has influenced contemporary treatment selection, while placing other targeted and conventional therapies within the broader evolving treatment landscape.

## Review

This narrative review synthesizes evidence from a PubMed/Medical Literature Analysis and Retrieval System Online (MEDLINE) search using terms related to TED, including disease activity and severity, clinical assessment, teprotumumab and other targeted therapies, conventional immunomodulatory treatments, orbital radiotherapy, DON, and orbital surgery. The search was supplemented by targeted retrieval of major clinical guidelines and consensus statements and by screening reference lists of key reviews and pivotal studies.

Priority was given to contemporary guidelines and consensus statements, randomized controlled trials, prospective clinical studies, and systematic reviews. Earlier publications were retained when they provided foundational evidence on disease pathogenesis, natural history, and established clinical assessment. Articles were selected based on clinical relevance to the objectives of this narrative review rather than through a formal systematic-review screening process; no quantitative synthesis or formal risk-of-bias grading was performed.

Pathogenesis of TED

The complex interplay of immune, inflammatory, and fibrotic mechanisms targeting orbital tissues contributes to TED through an autoantibody-mediated process. TSHR and IGF-1R are expressed by orbital fibroblasts. TSHR autoantibodies are central to Graves’ disease, while IGF-1R signaling can amplify orbital fibroblast activation and downstream inflammatory pathways [8-10].

Orbital fibroblasts play a key role in tissue remodeling and can differentiate into adipocytes and myofibroblasts under the influence of cytokines. This contributes to expansion of orbital fat and fibrosis associated with extraocular muscle dysfunction [8,9]. Pro-inflammatory cytokines, including interferon-gamma, tumor necrosis factor-alpha, and interleukin-1 beta, contribute to the inflammatory response and tissue remodeling. B cells contribute through antibody production, antigen presentation, and interactions with T cells [14]. Excessive hyaluronan production by orbital fibroblasts contributes to tissue edema and increased orbital volume [8,9].

TED typically follows a biphasic or evolving course, with an active inflammatory phase followed by a more stable inactive phase. The active phase may last months to years and can include progressive inflammation, pain, edema, proptosis, and visual symptoms. The inactive phase is characterized by reduced inflammatory activity but may leave persistent fibrosis, restrictive myopathy, proptosis, and disfigurement [1,2].

Activity and severity assessment

Activity Scoring

The Clinical Activity Score (CAS) is widely used to quantify inflammatory activity. The original seven-item CAS includes spontaneous orbital pain, pain on eye movement, eyelid erythema, eyelid swelling, conjunctival injection, chemosis, and inflammation of the caruncle or plica. A score of 3 or higher out of 7 is generally considered evidence of active disease [15]. Follow-up assessment should also consider changes in diplopia, visual acuity, soft-tissue findings, and proptosis. CAS is useful for identifying patients who may benefit from anti-inflammatory or targeted therapy but should be interpreted in the context of the overall phenotype.

Severity Classification

Severity reflects functional and structural impairment rather than inflammatory activity alone. Contemporary guidance generally categorizes TED as mild, moderate-to-severe, or sight-threatening [16,17]. Mild disease may involve limited eyelid changes, mild soft-tissue involvement, modest proptosis, and intermittent or absent diplopia without sight-threatening complications. Moderate-to-severe disease is characterized by more substantial proptosis, eyelid retraction, diplopia or restrictive myopathy, soft-tissue involvement, and a meaningful impact on daily life. Sight-threatening diseases include DON and severe corneal exposure or breakdown and require urgent treatment [16,17].

The Vision, Inflammation, Strabismus, and Appearance (VISA) framework provides another structured approach to documenting disease manifestations across four domains: vision, inflammation, strabismus, and appearance/exposure [18]. In practice, activity and severity should be assessed together because a patient may have severe but inactive structural disease or relatively modest structural disease with active inflammation.

Diagnostic evaluation

Assessment of thyroid function, including serum TSH and free thyroid hormone levels, is appropriate as part of the systemic evaluation of TED, with thyroid autoantibody testing considered particularly when the diagnosis is uncertain or thyroid dysfunction is not established [17]. Although TED remains primarily a clinical diagnosis, orbital imaging is valuable in atypical or asymmetric presentations, suspected DON, and surgical planning [2,5,17]. CT provides excellent assessment of extraocular muscle enlargement, orbital fat expansion, apical crowding, and bony anatomy and is particularly useful before orbital decompression, whereas MRI offers superior soft-tissue characterization and may provide additional information regarding inflammatory activity [2,5,17]. Advances in orbital imaging, including quantitative MRI approaches, may provide more objective characterization of orbital tissue involvement and disease activity, although their role in routine clinical practice continues to evolve [2,5,17].

Management of TED

Management is guided primarily by disease activity, severity, phenotype, and treatment goals. The following framework emphasizes first-line supportive and anti-inflammatory measures, targeted therapy, adjunctive treatment, and staged surgical rehabilitation, as summarized in Table 1.

**Table 1:** Clinical framework for assessment and management of thyroid eye disease according to disease activity and severity. TED: thyroid eye disease; CAS: Clinical Activity Score; DON: dysthyroid optic neuropathy; QOL: quality of life; RCTs: randomized controlled trials

Clinical state	Typical features	Treatment approach	Evidence basis	Important considerations
Mild, active or inactive	Limited lid/soft-tissue disease; no sight-threatening features	Lubrication, exposure control, smoking cessation, thyroid control; selenium in appropriate selenium-insufficient populations	RCT/guideline-supported for selected measures	Avoid unnecessary immunosuppression; monitor progression
Active moderate-to-severe	Inflammation with significant proptosis, diplopia, soft-tissue disease or QOL impact	IV glucocorticoids ± mycophenolate; targeted IGF-1R therapy; selected tocilizumab or rituximab; radiotherapy in selected phenotypes	RCTs/consensus/guidelines	Choice should reflect phenotype, contraindications, access, and treatment goal
Sight-threatening	DON and/or severe corneal exposure/breakdown	Urgent high-dose IV glucocorticoids; urgent decompression if inadequate response; aggressive corneal protection	Guideline/clinical evidence	Vision-threatening disease requires rapid escalation
Inactive residual disease	Stable proptosis, restrictive strabismus, lid retraction or exposure	Staged rehabilitative surgery	Established clinical practice	Decompression generally precedes strabismus surgery, followed by eyelid surgery
Active or longstanding/low-activity moderate-to-severe TED	Clinically significant proptosis, diplopia, inflammation or persistent disfiguring disease; chronic disease may have low CAS	Teprotumumab (IGF-1R inhibitor): 8 IV infusions every 3 weeks; consider phenotype, disease duration, comorbidities and goals	Multiple RCTs plus chronic/low-activity and retreatment evidence	Hearing impairment, hyperglycemia, muscle spasm; durability and retreatment remain important uncertainties

Mild TED and selenium supplementation

Patients with mild TED generally receive supportive treatment, particularly during the active phase. Measures include ocular lubrication, head-of-bed elevation, prisms when appropriate for diplopia, and management of ocular-surface disease. Smoking cessation is particularly important because smoking is a well-established modifiable risk factor [1,16]. Maintaining stable thyroid function is also important; antithyroid drugs may be used to control hyperthyroidism while restoring and maintaining euthyroidism [16,17].

Selenium supplementation may be beneficial for selected patients with mild TED, particularly in selenium-deficient populations. A randomized placebo-controlled European trial found improved quality of life and ocular outcomes and reduced progression with selenium 100 μg twice daily for six months [19]. Excessive or prolonged supplementation should be avoided because high selenium intake can cause adverse effects [20].

Radioactive iodine is an effective treatment for hyperthyroidism but can worsen TED in selected high-risk patients. Current guidance supports individualized use and consideration of glucocorticoid prophylaxis when the risk of TED progression is substantial [16,17].

Active moderate-to-severe TED

First-Line Anti-inflammatory Therapy

Intravenous glucocorticoids remain an important treatment for active moderate-to-severe TED, particularly when the immediate goal is to reduce inflammatory activity. Intravenous methylprednisolone is generally preferred to oral therapy because of greater efficacy and a more favorable tolerability profile. A commonly used regimen is a cumulative dose of 4.5 g administered over 12 weeks, although higher cumulative doses may be considered selectively. Cumulative dosing should be limited because of risks including hepatotoxicity, cardiovascular events, and infection [16,17].

Mycophenolate may be combined with intravenous glucocorticoids. The MINGO (mycophenolate sodium in Graves' orbitopathy) randomized trial demonstrated greater overall response with combination therapy than glucocorticoid monotherapy in active moderate-to-severe TED [21]. Appropriate monitoring for gastrointestinal effects, myelosuppression, and infection is required.

Teprotumumab: From Mechanism to Clinical Positioning

Teprotumumab is a fully human monoclonal antibody targeting the IGF-1R, a pathway that interacts with TSHR signaling and contributes to orbital fibroblast activation, glycosaminoglycan production, adipogenesis, and tissue expansion [9,10]. Its clinical importance lies not simply in demonstrating efficacy but in challenging the traditional assumption that treatment benefit is confined to highly active, early TED. The standard regimen consists of an intravenous infusion of 10 mg/kg for the first dose followed by 20 mg/kg every three weeks for seven additional infusions, for a total of eight infusions over approximately 21 weeks [22,23]. The phase 2 trial established proof of concept, with 69% of teprotumumab-treated patients achieving the composite response of at least a two-point reduction in CAS and at least a 2-mm reduction in proptosis at week 24, compared with 20% with placebo [10]. The phase 3 OPTIC (Treatment of Graves’ Orbitopathy (Thyroid Eye Disease) to Reduce Proptosis with Teprotumumab Infusions in a Randomized, Placebo-Controlled, Clinical Study) trial subsequently confirmed a robust proptosis response: 83% versus 10% of placebo-treated patients achieved at least a 2-mm reduction, while improvements were also demonstrated in diplopia, CAS, and Graves’ ophthalmopathy-quality of life (GO-QOL) questionnaire [22]. Because these pivotal trials enrolled patients with relatively recent and active disease, their results initially supported teprotumumab primarily as therapy for active moderate-to-severe TED. Subsequent evidence has broadened that interpretation. In a randomized placebo-controlled trial of patients with TED of two to 10 years' duration and low disease activity, teprotumumab produced a mean proptosis improvement of 2.41 mm versus 0.92 mm with placebo at week 24 (between-group difference, 1.48 mm; 95% CI, 0.69-2.28; P=.0004) [11]. This finding is clinically important because it demonstrates that treatment response can occur despite prolonged disease duration and minimal inflammatory activity. It suggests that persistent IGF-1R-related orbital remodeling may remain therapeutically relevant after the conventionally defined active phase. Treatment timing also deserves reconsideration. A post hoc analysis found that absence of a substantial proptosis response by week 12 did not necessarily predict treatment failure: 63% of patients who had not achieved a 2-mm proptosis reduction at week 12 subsequently achieved that response by week 24 [24]. Thus, early response kinetics should not automatically be used to discontinue a complete treatment course. Retreatment represents another evolving aspect of teprotumumab use. OPTIC-X (Treatment of Graves’ Orbitopathy to Reduce Proptosis with Teprotumumab Infusions in an Open-Label Clinical Extension Study) demonstrated that patients with longer-duration disease could respond to treatment and that selected patients with inadequate initial response or subsequent flare could benefit from retreatment [12]. More recent multicenter real-world data from 119 patients found a 24% retreatment rate within one year after a complete initial course; among those retreated, 82% had demonstrated a clinically significant proptosis response during the initial course [25]. These observations support retreatment as a clinically relevant strategy, but they do not establish an optimal retreatment schedule or identify which patients should receive it. Durability remains one of the most important unresolved questions. In pooled long-term follow-up of three clinical trials, responses in proptosis, diplopia, disease activity, and quality of life were assessed through week 72, providing evidence that clinically meaningful benefits can persist after the treatment course in many patients [26]. However, later observational studies have raised concerns about regression. In one cohort, 66.4% of eyes demonstrated some proptosis regression from the immediate post-treatment measurement at a mean of 20.4 months, although 60.6% of eyes still had net improvement compared with pretreatment [27]. An extended follow-up of the same cohort found that 67.2% of eyes continued to worsen after initial regression and that 54.0% of patients experienced a TED flare [28]. These findings argue against equating an excellent short-term response with permanent disease modification. The distinction is important: teprotumumab clearly modifies clinical manifestations, but whether a standard eight-infusion course permanently changes the underlying trajectory of TED remains uncertain. Safety considerations are increasingly informed by postmarketing and longitudinal data. Muscle spasm, nausea, diarrhea, fatigue, alopecia, hearing impairment, and hyperglycemia are among the recognized adverse effects [29,30]. Hearing dysfunction may occur during treatment and can be reversible or persistent in some patients; prospective audiometry studies support baseline and follow-up auditory assessment, particularly in patients with pre-existing auditory risk [31]. Glycemic effects are usually manageable but warrant particular attention in patients with diabetes or prediabetes. In pooled clinical-trial data, hyperglycemic episodes occurred predominantly in patients with baseline dysglycemia, and regular glucose monitoring allowed most events to be managed medically [30]. More recent longitudinal data suggest that glycemic abnormalities may persist beyond treatment in some patients, supporting continued follow-up rather than monitoring only during the infusion period [32]. The current U.S. prescribing information indicates that TEPEZZA is approved for TED regardless of disease activity or duration and recommends 10 mg/kg initially followed by 20 mg/kg every three weeks for seven additional infusions [23]. The optimal place of teprotumumab within treatment algorithms remains phenotype-dependent. Its strongest evidence concerns reduction of proptosis and improvement in diplopia, but treatment selection should also consider disease duration, inflammatory activity, optic nerve status, extraocular muscle restriction, previous therapy, comorbidities, patient priorities, cost, and access. In patients with sight-threatening disease, urgent decompression may remain necessary when vision is threatened, and medical treatment is inadequate; teprotumumab should not delay sight-saving intervention. Conversely, in patients with persistent disfiguring proptosis or diplopia who have low inflammatory activity but no immediate threat to vision, the evidence in longstanding disease has expanded the rationale for considering targeted therapy before proceeding directly to rehabilitative surgery [11,17]. Other targeted immunomodulators remain relevant but occupy a more selective position. Tocilizumab, an IL-6 receptor antagonist, has demonstrated reductions in disease activity in corticosteroid-resistant active TED and may be considered when conventional therapy is inadequate or contraindicated [33,34]. Rituximab, which depletes CD20-positive B cells, has a biologically plausible role but less consistent efficacy across trials and patient populations [35]. These agents are therefore best viewed as alternatives or rescue therapies in selected phenotypes rather than equivalent competitors to teprotumumab. Veligrotug, another IGF-1R-directed antibody, provides important proof that the therapeutic relevance of IGF-1R inhibition extends beyond a single molecule. In the phase 3 THRIVE trial, a 12-week course produced rapid improvements in proptosis, diplopia, and disease activity, with 70% of initial responders maintaining proptosis response at week 52 [13]. Comparative studies will be needed to determine whether differences in molecular characteristics, dosing, durability, safety, or access translate into clinically meaningful advantages among IGF-1R-directed therapies. Taken together, teprotumumab should not be viewed simply as another immunosuppressive drug. Its most important contribution has been to broaden the therapeutic window of TED: benefit can occur in highly active disease but also in longstanding disease with low activity. At the same time, retreatment, relapse, proptosis regression, and long-term safety demonstrate that the treatment course is not necessarily synonymous with permanent disease modification. Future studies should therefore move beyond short-term responder rates and examine predictors of durable benefit, optimal retreatment strategies, comparative effectiveness, biomarkers of response, and whether earlier or phenotype-specific intervention can alter the long-term course of TED. 

Key quantitative findings from pivotal and recent studies of IGF-1R-directed therapy are summarized in Table 2.

**Table 2:** Selected quantitative evidence from pivotal and recent targeted-therapy studies in TED. TED: thyroid eye disease; CAS: Clinical Activity Score; OPTIC-X: Treatment of Graves’ Orbitopathy to Reduce Proptosis with Teprotumumab Infusions in an Open-Label Clinical Extension Study; QOL: quality of life; IGF-1R: insulin-like growth factor-1 receptor

Study	Population/design	Intervention	Key quantitative finding	Clinical interpretation
Smith et al., 2017 [10]	Active, moderate-to-severe TED; randomized placebo-controlled trial (n=88)	Teprotumumab; 8 IV infusions every 3 weeks	Primary response at week 24: 69% (29/42) vs 20% (9/45) with placebo; P<0.001	Established proof of efficacy for active disease, including combined proptosis and CAS response.
Douglas et al., 2020 [22]	Active, moderate-to-severe TED; randomized placebo-controlled phase 3 trial (n=83)	Teprotumumab; 8 IV infusions every 3 weeks	Proptosis response at week 24: 83% (34/41) vs 10% (4/42); mean proptosis change −2.82 vs −0.54 mm; P<0.001	Confirmed robust effects on proptosis, activity, diplopia, and QOL.
Douglas et al., 2022 (OPTIC-X) [12]	OPTIC participants previously receiving placebo, plus teprotumumab nonresponders or responders with flare	Teprotumumab; treatment or retreatment	Previously placebo-treated patients: 89.2% (33/37) became proptosis responders; 62.5% (5/8) of selected patients with flare responded to retreatment	Supports efficacy in longer-duration TED and provides evidence for selected retreatment.
Douglas et al., 2023 [11]	TED duration 2–10 years; low disease activity; randomized placebo-controlled trial (n=62)	Teprotumumab; 8 IV infusions every 3 weeks	Mean proptosis improvement at week 24: 2.41 mm vs 0.92 mm; between-group difference 1.48 mm (95% CI 0.69–2.28); P=.0004	Challenges a strict inflammation- or duration-based therapeutic window.
Yen et al., 2026 (THRIVE) [13]	Active, moderate-to-severe TED; phase 3 randomized placebo-controlled trial (n=113)	Veligrotug; 5 IV infusions over 12 weeks	At week 15: proptosis response 70% vs 5%; mean Hertel proptosis reduction 2.90 vs 0.48 mm; at week 52, 70% of initial responders maintained response	Supports IGF-1R inhibition as a broader therapeutic strategy; comparative evidence and regulatory availability continue to evolve.

Adjunctive therapy: orbital radiotherapy

Orbital radiotherapy may be useful in selected patients with active moderate-to-severe TED, particularly when restrictive myopathy and diplopia are prominent. A commonly used regimen is 20 Gy delivered in 10 fractions. Its greatest effect is generally on ocular motility and diplopia, while effects on proptosis and soft-tissue swelling are more modest [36]. Potential complications include radiation retinopathy and optic neuropathy, particularly in patients with pre-existing microvascular disease.

Sight-threatening TED

DON is an ophthalmic emergency because of the risk of irreversible visual loss. High-dose intravenous glucocorticoids are first-line treatment, with close monitoring of visual acuity, color vision, pupillary function, visual fields, and other indicators of optic nerve function. If the response to medical therapy is inadequate, urgent orbital decompression may be required [5,37].

Severe exposure keratopathy with corneal breakdown is another sight-threatening manifestation. Treatment may include intensive lubrication, ocular-surface protection, topical antimicrobial therapy when indicated, tarsorrhaphy, amniotic membrane transplantation, botulinum toxin injection, eyelid procedures, and orbital decompression in selected patients with substantial proptosis [38].

Surgical rehabilitation

Surgical rehabilitation is generally performed during the inactive phase once orbital inflammation and clinical measurements have stabilized. Surgery may be indicated for persistent disfiguring proptosis, restrictive diplopia, eyelid malposition, or ocular-surface exposure [16,17].

When more than one rehabilitative procedure is required, orbital decompression is generally performed first, followed by strabismus surgery after ocular alignment has stabilized, with eyelid surgery performed last because decompression and extraocular muscle surgery can alter eyelid position [16,17]. Surgical planning should be individualized according to disease manifestations, functional impairment, patient expectations, and comorbidities. CT or MRI can assist planning, particularly in patients with suspected compressive optic neuropathy.

## Conclusions

TED is a complex autoimmune orbital disorder with variable clinical expression and therapeutic requirements. Accurate assessment of both disease activity and severity is central to individualized management. Activity reflects ongoing inflammatory biology and potential responsiveness to medical therapy, whereas severity captures the functional and anatomical burden of disease.

Contemporary management increasingly integrates supportive care, risk-factor modification, intravenous glucocorticoids, mycophenolate, targeted biologic therapy, selected orbital radiotherapy, and staged surgical rehabilitation. Teprotumumab deserves particular emphasis because it has broadened therapeutic options beyond the traditional paradigm of treating predominantly early, highly active disease. Emerging evidence in longstanding and less-active TED, together with data on delayed response, retreatment, durability, and reactivation, suggests that the therapeutic window may be broader and more complex than previously recognized. At the same time, questions regarding long-term safety, cost, access, and appropriate patient selection remain important.

Future research should clarify the extent to which teprotumumab and other IGF-1R-directed therapies provide sustained clinical benefit and whether their effects represent durable alteration of disease biology or prolonged control of established orbital manifestations. Comparative effectiveness, optimal retreatment strategies, predictors of response, biomarkers, cost-effectiveness, and equitable access remain important unanswered questions. Overall, evolving evidence supports an individualized approach to TED treatment based on activity, severity, disease duration, phenotype, and the patient's principal therapeutic goal, rather than disease activity alone.
